# Melusin Promotes a Protective Signal Transduction Cascade in Stressed Hearts

**DOI:** 10.3389/fmolb.2016.00053

**Published:** 2016-09-12

**Authors:** Matteo Sorge, Mara Brancaccio

**Affiliations:** Department of Molecular Biotechnology and Health Sciences, University of TorinoTorino, Italy

**Keywords:** chaperone, Melusin, intracellular signaling, apoptosis, ERK 1/2, AKT, HSP90, heart failure

## Abstract

Melusin is a chaperone protein selectively expressed in heart and skeletal muscles. Melusin expression levels correlate with cardiac function in pre-clinical models and in human patients with aortic stenosis. Indeed, previous studies in several animal models indicated that Melusin plays a broad cardioprotective role in different pathological conditions. Chaperone proteins, besides playing a role in protein folding, are also able to facilitate supramolecular complex formation and conformational changes due to activation/deactivation of signaling molecules. This role sets chaperone proteins as crucial regulators of intracellular signal transduction pathways. In particular Melusin activates AKT and ERK1/2 signaling, protects cardiomyocytes from apoptosis and induces a compensatory hypertrophic response in several pathological conditions. Therefore, selective delivery of the Melusin gene in heart via cardiotropic adenoviral associated virus serotype 9 (AAV9), may represent a new promising gene-therapy approach for different cardiac pathologies.

## Role of chaperones and co-chaperones in the stressed heart

Proteins are synthesized as linear amino acid chains that must fold in a specific three-dimensional structure and maintain their functional conformation to carry out their biological functions (Balchin et al., 2016). However, the preservation of a particular fold depends on different factors, including temperature, pH, protein-protein interactions, post-translational modifications, mechanical stretch, etc. Moreover, in several cases, proteins need to change conformation to accomplish their functions. For instance, extracellular ligands, by binding to membrane receptors, are able to trigger a cascade of conformational changes in cytoplasmic signal transduction proteins. During structural switches, proteins pass through metastable intermediates exposing hydrophobic amino acid residues, potentially causing toxic protein aggregates. Cells evolved an organized chaperone system to cope with misfolding of native proteins and assist physiological protein conformational changes and unfolding emergencies.

The highly specialized sarcomeric structures in cardiomyocytes consist of a number of proteins bound to each other in a very regulated fashion, creating a dense protein matrix. Mechanical overload is sensed by membrane receptors connecting the extracellular matrix to the intracellular cytoskeleton, like integrins (Brancaccio et al., 2006). Growing evidence indicates that excessive mechanical stretch, such as the ones induced by hypertension, aortic stenosis, myocardial infarction, etc., induces protein misfolding (Willis and Patterson, 2010; Tarone and Brancaccio, 2014; McLendon and Robbins, 2015; Parry et al., 2015). Moreover, mechanical stretch and humoral factors released in response to excessive workload activate signal transduction pathways (Tarone and Lembo, 2003) that need to be assisted by chaperones to properly sustain cell survival and induce cardiomyocyte hypertrophic growth. Unfolded proteins, by exposing hydrophobic amino acid stretches, are prone to form insoluble toxic aggregates in cardiomyocytes, potentially contributing to cell death (Del Monte and Agnetti, 2014; Parry et al., 2015). Accordingly, patients suffering from hypertrophic cardiomyopathy and idiopathic dilated cardiomyopathy accumulate misfolded proteins in the heart (Parry et al., 2015).

Chaperone proteins are characterized by different molecular weight, subcellular localization and enzymatic activity. In response to stressful conditions, chaperone expression is induced in cardiomyocytes to cope with the unfolding emergency. However, if stress conditions persist, chaperone activity becomes insufficient for protecting cells form proteotoxicity, and pathological remodeling takes place (Del Monte and Agnetti, 2014). Several chaperones have been described to build a compensatory response in the stressed heart in a cooperative manner (Willis and Patterson, 2010; Tarone and Brancaccio, 2014). HSP90 is one of the most important molecular chaperones, acting as a dynamic dimer that switches through multiple conformations. Co-chaperones regulate HSP90 ATPase activity, interact with further components of the machinery and drive substrate binding (Li et al., 2012; Verma et al., 2016). HSP90 interacts with a variety of substrates also called “HSP90 client proteins” among them transcription factors, signaling molecules, apoptosis regulators, and cytoskeletal components (http://www.picard.ch/downloads/downloads.htm), controlling their activity and degradation. It is thus conceivable that Hsp90 plays multiple important roles in sustaining heart function upon stress adaptation, by inducing protein refolding, directing unfolded proteins to proteasome degradation, and assisting conformational changes in signal transduction molecules (Ficker et al., 2003; Kupatt et al., 2004; Tarone and Brancaccio, 2014; Parry et al., 2015).

A second class of chaperones, the small heat shock proteins (sHsps) family, is devoid of ATPase activity and characterized by an α-crystallin domain responsible for their oligomerization. The association between sHsp oligomers and client proteins is required for their anti-aggregation activity (Vos et al., 2011; Bakthisaran et al., 2015; Haslbeck and Vierling, 2015). sHsps show anti-apoptotic activity, inhibit misfolded protein aggregation, mediate protein refolding in cooperation with Hsp90, and regulate signal transduction pathways (Vos et al., 2008, 2011; Bakthisaran et al., 2015). Growing experimental evidence indicates a role for this class of chaperones in sustaining heart function in stress conditions (Willis and Patterson, 2010; Tarone and Brancaccio, 2014; Parry et al., 2015). For instance, αB-crystallin protects the heart from ischemia/reperfusion injury and, when overexpressed, it attenuates cardiac hypertrophy caused by pressure overload. Moreover, missense mutations in the αB-crystallin coding gene cause a desmin related cardiomyopathy (Boelens, 2014; Anbarasu and Sivakumar, 2016). Hsp27, another chaperone expressed in the heart, protects from ischemia/reperfusion damage when overexpressed (Christians et al., 2012). Hsp20 also displays a well-documented cardioprotective activity by enhancing cardiomyocyte survival and improving heart contractility in different models of heart failure (Fan and Kranias, 2011; Martin et al., 2014).

In this review we will focus on the cardioprotective role of the muscle specific chaperone protein Melusin, showing both Hsp90 co-chaperone function and typical features of sHsps.

## Melusin structure and chaperone function

Melusin is a chaperone protein, encoded by the *ITGB1BP2* gene, expressed selectively in heart and skeletal muscles. Melusin has been identified as an interactor of the cytoplasmic region of β1 integrin (Brancaccio et al., 1999), a membrane receptor that connects the intracellular cytoskeleton with the extracellular matrix, allowing muscle cells to respond to mechanical stimuli (Brancaccio et al., 2006). This chaperone protein shows a highly conserved structure in vertebrates, consisting of two Cysteine and Histidine-Rich Domains (CHORDS), a CS domain, shared by CHORD proteins and by the co-chaperone protein Sgt1 (Shirasu et al., 1999), and a C-terminal Ca^2+^-binding domain, enriched in aspartic and glutamic acid residues (Brancaccio et al., 1999). CHORD I-II domains in the amino-terminal region of Melusin are 60-amino acid zinc-binding domains, highly conserved during evolution and able to mediate the binding of Melusin to HSP90 (Hahn, 2005; Sbroggiò et al., 2008; Hong et al., 2013). Moreover, the CS domain, structurally similar to α-crystallin and p23 chaperone proteins (Garcia-Ranea et al., 2002), has also been described as an HSP90 binding module (Lee et al., 2004; Zhang et al., 2010). Melusin, through its CHORD domains, directly binds the ATPase domain of HSP90 (Sbroggiò et al., 2008) preferentially in its ADP-bound state (Gano and Simon, 2010; Zhang et al., 2010; Hong et al., 2013). In addition, the binding of Ca^2+^ to the C-terminal domain of Melusin enhances its interaction with HSP90 (Hong et al., 2013). This is particularly relevant considering the crucial role of Ca^2+^ ions in muscle contraction and the link between heart failure and Ca^2+^ cycling dysfunction in cardiomyocytes (Marks, 2013). Interestingly, Melusin inhibits denatured protein aggregation *in vitro* in a dose dependent manner, showing a potential intrinsic chaperone activity (Sbroggiò et al., 2008), and displays the ability to oligomerize (Hong et al., 2013), a feature correlated with increased chaperone activity in small heat shock proteins (Garrido et al., 2012; Bakthisaran et al., 2015; Haslbeck and Vierling, 2015). These are relevant functions considering the accumulation of toxic misfolded proteins occurring during cardiomyopathy (Willis and Patterson, 2013; Del Monte and Agnetti, 2014; Tarone and Brancaccio, 2014). However, it is noteworthy that the ability of Melusin to act as a chaperone is based only on *in vitro* experiments and further evidences are required to confirm this property *in vivo*.

## Melusin cardioprotective role in animal models of cardiomyopathy

The protective role of Melusin in the heart is strictly related to the cardiac response to stress stimuli. Upon mechanical stretch, the heart activates a compensatory hypertrophic response, causing an increase in the thickness of the left ventricle wall that preserves contractility. However, if the stimulus becomes chronic, the heart undergoes a pathological evolution from adaptive hypertrophy to dilated cardiomyopathy with loss of contractile function, known as “maladaptive remodeling.”

In a mouse model subjected to cardiac mechanical stretch via surgical aortic banding, mimicking human pathologies such as chronic aortic stenosis, left ventricle outflow obstruction, or systemic hypertension, Melusin expression levels increase in left ventricles in the first week of pressure overload, during the induction of the compensatory hypertrophic response (De Acetis et al., 2005; Sbroggiò et al., 2008). However, Melusin expression decreases when chamber dilation and loss of contractility ensues (De Acetis et al., 2005). Notably, this correlation between Melusin expression levels and cardiac response to stress has been reported also in a dog model of volume overload (Donker et al., 2007). Another pathological condition characterized by maladaptive remodeling in humans is the myocardial infarction (Heusch et al., 2014). The occlusion of coronary arteries induces an ischemic insult to the cardiac tissue in which the damaged area is replaced by a non-contractile connective scar and the healthy portion undergoes hypertrophy because of increased hemodynamic stress. In a rat model of myocardial infarction obtained by permanent coronary ligation, analysis at different time points after the ischemic injury revealed a direct correlation between Melusin expression and the rates of left ventricle systolic pressure and fractional shortening (Gu et al., 2012).

To investigate the specific role of Melusin in protecting myocardium from adverse remodeling *in vivo*, Melusin-*null* mice (Brancaccio et al., 2003) and transgenic mice overexpressing Melusin in cardiomyocytes (De Acetis et al., 2005) have been generated. Melusin-*null* mice are healthy and fertile under normal conditions and they do not display obvious defects in striated muscle development and structure. These mice show normal myocardial parameters (Brancaccio et al., 2003), indicating that Melusin is not crucial for cardiac development and basal physiological functions. However, the cardioprotective role of Melusin becomes evident under mechanical stress conditions. Whereas wild-type mice activate a compensatory hypertrophic response after 4 weeks following aortic stenosis, Melusin-*null* mice fail to activate this program and rapidly develop a dilated cardiomyopathy with left ventricle dilation, chamber wall thinning, and impaired contractility (Brancaccio et al., 2003).

The importance of Melusin in the cardiac response to mechanical stretch has been further confirmed by analyzing Melusin overexpressing mice. In basal conditions, these mice show a mild cardiomyocyte hypertrophy without alteration of contractile function (De Acetis et al., 2005). Instead, upon pressure overload conditions, Melusin overexpression effectively protects mouse myocardium by sustaining the compensatory hypertrophic response and healthy contractile function even after 12 weeks of aortic banding, when wild-type mice have already developed a dilated cardiomyopathy and heart failure (De Acetis et al., 2005).

**Figure 1:**
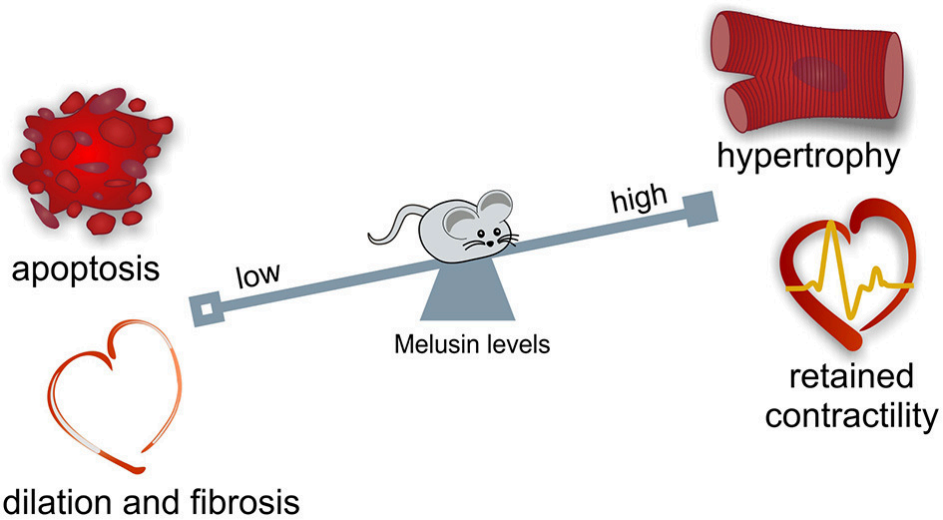
**Melusin cardioprotective role in the heart**. Melusin protects the heart from “maladaptive remodeling” during mechanical stress. Melusin potentiates the compensatory hypertrophic response of the heart preserving its contractile function. Melusin also prevents cardiac dilation and fibrosis deposition and protects cardiomyocytes from apoptotic death.

During maladaptive remodeling of the heart, cardiomyocyte loss, inflammation, deposition of fibrotic tissue, and reduction of capillary density typically occur. Notably, Melusin overexpression protects cardiomyocytes from apoptotic death, reduces inflammation and stromal tissue deposition and stimulates capillary growth (De Acetis et al., 2005; Figure 1).

In mice overexpressing Melusin in which myocardial infarction is induced by permanent left descending coronary ligation, Melusin ensures a double protective role. In the early phase, it reduces the inflammatory response and protects against cardiac rupture; in long term recovery it prevents the heart from dilated degeneration by improving cardiomyocyte survival (Unsold et al., 2014).

Upon acute coronary occlusion, the duration of the ischemia determines the extent of cardiac damage. In clinical practice, acute myocardial infarction is treated with angioplasty or thrombolysis to induce a prompt coronary re-opening. However, sudden re-oxygenation determines the so-called “reperfusion injury,” characterized by the production of reactive oxygen species, calcium overload, and the opening of the mitochondrial permeability transition pore, which all cause cardiomyocyte death and subsequent inflammation, thereby worsening the initial ischemic damage (Yellon and Hausenloy, 2007; Perrelli et al., 2011). The ischemia-reperfusion injury is well-reproduced in isolated perfused hearts with the Langendorff technique. In this model, Melusin overexpression significantly reduces the infarct size area and cardiomyocyte cell death, thus protecting the heart from ischemia-reperfusion injury (Penna et al., 2014).

## Melusin in human cardiomyopathies

A link between Melusin expression and cardiac functional parameters has also been observed in humans. In a cohort of 17 patients with aortic stenosis evolved to severe heart failure, Melusin expression positively correlates with left ventricle ejection fraction (Brokat et al., 2007). A genetic screening for Melusin mutations in cardiomyopathic patients, performed by three independent laboratories, have revealed two missense mutations, a His13Tyr mutation in the CHORD I domain in a family with hypertrophic cardiomyopathy (Palumbo et al., 2009), and an Ala313Gly mutation in the carboxy terminal region linking the CS domain to the acidic domain in a family with dilated cardiomyopathy (Ruppert et al., 2013) but their segregation in the family members not always correlates with the onset of cardiomyopathy, making their causative significance unclear. Furthermore, structural analysis on the Melusin homolog in plants suggests that His13Tyr mutation does not disrupt the interaction with Hsp90 and that Ala313Gly mutation is distant from known CS domain interaction sites (Zhang et al., 2010). Another silent mutation, an intronic duplication and some polymorphisms has also been found in a study of population screening comparing cardiopathic and healthy subjects (Palumbo et al., 2009). However, all these analyses led to the consideration that Melusin gene (*ITGB1BP2*) mutations are very rare within the population, with no significant relevance in the epidemiology of cardiomyopathy. On the other hand, pre-clinical studies indicate that the regulation of Melusin expression may play a key role in improving the ability of the myocardium to cope with different stressors.

## Melusin cardioprotective signal transduction

Mechanical stress induces in the heart the activation of specific signal transduction pathways and the release of neurohumoral mediators acting on cardiomyocytes, fibroblasts and endothelial cells, regulating the heart's response to stress. The balance between these signals may direct the overall cardiac response to a compensatory or to a maladaptive remodeling (Tarone and Lembo, 2003). Extensive data indicate that the activation of the MAPK and AKT signal transduction pathways in cardiomyocytes promotes cell survival and compensatory hypertrophic growth, protecting the heart from dilation and failure (Selvetella et al., 2004; Baines and Molkentin, 2005; Tarone et al., 2013). Molecular analysis of the myocardial signaling pathways, indicate that Melusin interacts with several signaling molecules, including the Focal Adhesion Kinase (FAK), the MAPK scaffold protein IQGAP1, the mitogen activated protein kinases c-Raf, MEK1/2, and ERK1/2 (Sbroggiò et al., 2011a) and the phosphoinositide 3-kinases (PI3Ks), which in turn activate AKT (Waardenberg et al., 2011). We demonstrated a role for the Focal adhesion kinase in activating Melusin-bound ERK1/2 in response to mechanical stretch (Sbroggiò et al., 2011a). The Melusin binding protein IQGAP1 is a scaffold protein able to bind c-Raf, MEK1/2, and ERK1/2 and to facilitate their sequential phosphorylation. Accordingly, IQGAP1 is essential for the activation of the MAPK pathway in response to Melusin overexpression and pressure overload *in vivo* (Sbroggiò et al., 2011a,b).

**Figure 2:**
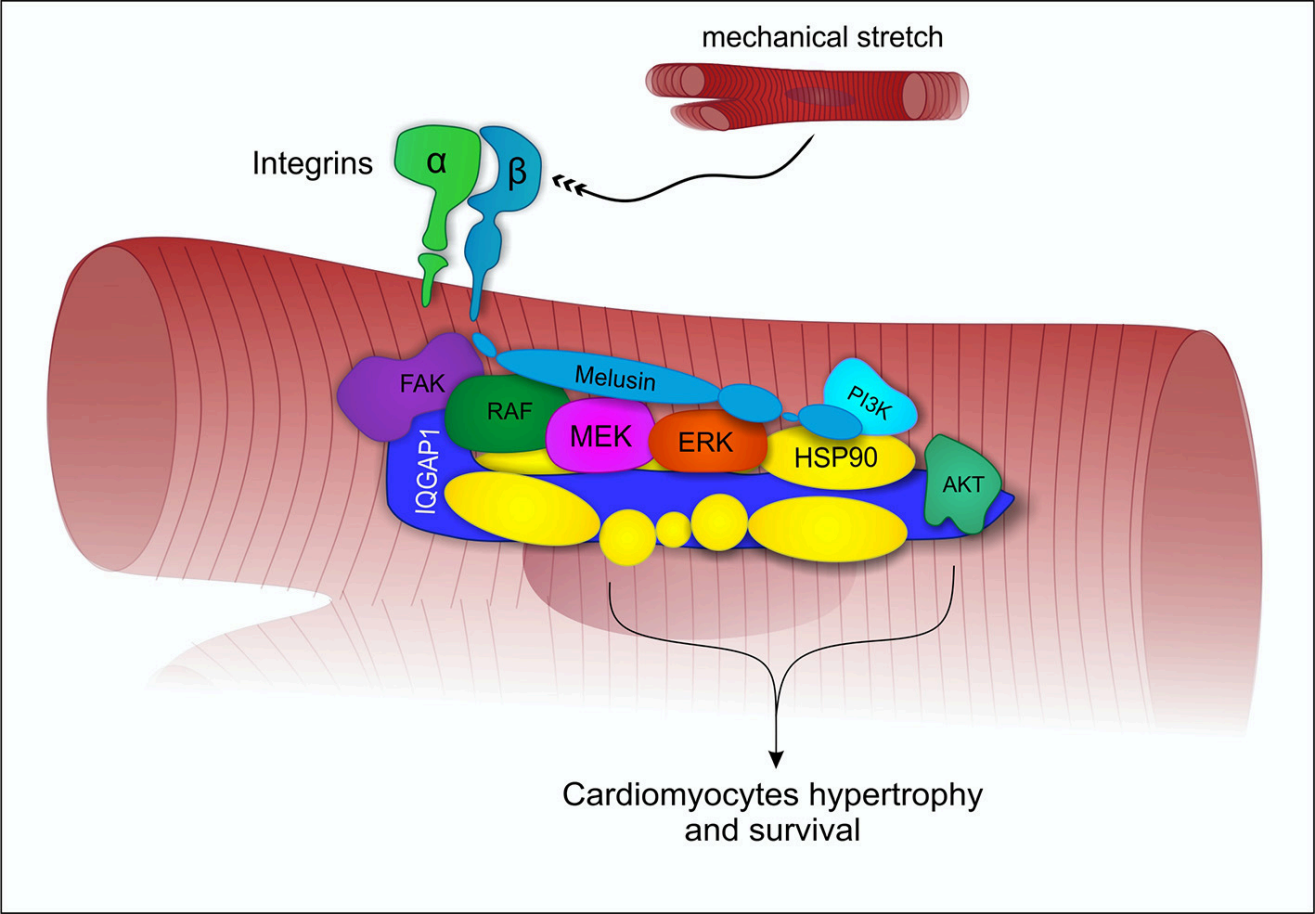
**Melusin-dependent activation of ERK1/2 and AKT under mechanical stress**. Under mechanical stretch condition, Melusin binds to the cytoplasmic domain of β1-integrin and interacts with IQGAP1, leading to the activation of ERK1/2 and AKT cardioprotective pathways.

These data suggest that Melusin, along with Hsp90, mediates the assembly of a signalosome organized on the scaffold protein IQGAP1 to activate and integrate beneficial ERK1/2 signaling (Tarone et al., 2013) with the AKT pathway (Figure 2). In accordance, a role for Hsp90 in promoting and maintaining the assembly of protein complexes has been previously described (Makhnevych and Houry, 2012).

Melusin, indeed, potentiates ERK1/2 and AKT phosphorylation in transgenic mice subjected to transverse aortic constriction, preventing the evolution to dilated cardiomyopathy (Brancaccio et al., 2003; De Acetis et al., 2005). In the model of myocardial infarction due to permanent coronary ligation, Melusin overexpression significantly enhances ERK1/2 phosphorylation at the myocardial infarct border zone, where mechanical stretch is higher, reducing the extent of the damage (Unsold et al., 2014). Furthermore, the activation of the reperfusion injury salvage kinase (RISK) pathway, composed by the PI3K/AKT and the ERK1/2 pathways, has been shown to protect cardiomyocytes during the reperfusion phase (Hausenloy and Yellon, 2004). This pathway is required for Melusin protective role in the ischemia/reperfusion model, since their pharmacological inhibition abrogates Melusin protection (Penna et al., 2014). Notably, in the pathological heart, when Melusin expression is decreased, also the ERK1/2 and AKT compensatory pathways are downregulated, allowing the establishment of detrimental signaling that further impact on heart function (De Acetis et al., 2005).

## Conclusion and therapeutic perspectives

Melusin is a chaperone protein selectively expressed in cardiac and skeletal muscles, indicating a tissue specific function for this protein. Accordingly, its expression is upregulated in response to mechanical overload in the heart during the hypertrophic phase (De Acetis et al., 2005; Sbroggiò et al., 2008). Melusin protective activity in these conditions has been widely demonstrated in a number of preclinical models (Brancaccio et al., 2003; De Acetis et al., 2005; Penna et al., 2014; Unsold et al., 2014) and associated with its ability to build a supramolecular complex activating ERK1/2 and AKT beneficial pathways in cardiomyocytes (Sbroggiò et al., 2011a,b). In this view, increasing Melusin expression in the heart may represent a new therapeutic approach in cardiomyopathic patients. To date, adeno-associated viruses (AVVs) are the most promising viral vectors for gene therapy, also approved by FDA for human clinical trials. AAVs ensure a stable and efficient transgene expression even in non-proliferating cells and, once in the human body, do not induce a significant immune response or insertional mutagenesis risk (Ponnazhagan et al., 1997; Chirmule et al., 1999). In particular adenoviral associated virus serotype 9 (AAV9) is able to direct transgene expression in cardiomyocytes, representing a promising vector for gene therapy in cardiac diseases. In our laboratory, an AAV9 virus carrying human Melusin cDNA, able to induce Melusin expression selectively in the heart is currently under investigation for its ability to counteract cardiomyopathy in different mouse models.

## Author contributions

MS wrote the manuscript and drew the figures. MB wrote the manuscript.

### Conflict of interest statement

The authors declare that the research was conducted in the absence of any commercial or financial relationships that could be construed as a potential conflict of interest.
